# Human papillomavirus genotype distribution and factors associated among female sex workers in West Africa

**DOI:** 10.1371/journal.pone.0242711

**Published:** 2020-11-25

**Authors:** Fatoumata Korika Tounkara, Ibrahima Téguété, Fernand A. Guédou, Ella Goma-Matsétsé, Amadou Koné, Luc Béhanzin, Sidy Traoré, Marlène Aza-Gnandji, Bintou Keita, Julie Guenoun, François Coutlée, Michel Alary

**Affiliations:** 1 Département de Médecine Sociale et Préventive, Université Laval, Québec, QC, Canada; 2 Axe Santé des Populations et Pratiques Optimales en Santé, Centre de Recherche du CHU de Québec—Université Laval, Québec, QC, Canada; 3 Centre Hospitalier Universitaire Gabriel Toure, Bamako, Mali; 4 Centre de Santé de Cotonou-1, Dispensaire des Infections Sexuellement Transmissibles (DIST), Cotonou, Benin; 5 Centre Universitaire de Recherche Clinique (UCRC), Bamako, Mali; 6 Des Techniques et Des Technologies de Bamako, Université des Sciences, Bamako, Mali; 7 Association de Recherche de Communication et d’Accompagnement à Domicile de personnes Vivant avec le VIH (ARCAD-SIDA), Bamako, Mali; 8 Département de Microbiologie et Infectiologie, Centre de Recherche du Centre Hospitalier de l’Université de Montréal, Montréal, QC, Canada; 9 Institut National de Santé Publique du Québec, Québec, QC, Canada; Rudjer Boskovic Institute, CROATIA

## Abstract

**Objectives:**

This study aimed to: (1) Estimate HPV prevalence and genotype distribution among female sex workers (FSWs) in Mali and Benin as well as the prevalence of multiple HPV type infections in this group, and (2) Identify potential risk factors associated with high-risk (HR) HPV infections.

**Methods:**

We analyzed baseline data of 665 FSWs aged ≥ 18 years recruited during a prospective cohort of cervical cancer screening in Cotonou (Benin) and Bamako (Mali) from 2017 to 2018. The Linear Array HPV genotyping test was used to identify HPV genotypes. Descriptive statistics and multivariate log-binomial regression were used. Adjusted prevalence ratios (APR) with 95% confidence intervals (95%CI) were estimated to identify risk factors associated with HR-HPV infections.

**Results:**

HPV data were available for 659 FSWs (Benin: 309; Mali: 350). The mean age was 35.0 years (± 10.7) in Benin and 26.8 years (± 7.6) in Mali. The overall HPV prevalence rates were 95.5% in Benin and 81.4% in Mali. About 87.7% and 63.4% of FSWs harbored ≥ 2 HPV types in Benin and Mali, respectively. The top three prevalent HR-HPV among FSWs in Benin were: HPV58 (37.5%), HPV16 (36.6%) and HPV52 (28.8%). Corresponding patterns in Mali were HPV16 (15.7%), HPV51 (14.3%) and HPV52 (12.9%). In Benin, the main factors associated with HR-HPV were vaginal douching (APR = 1.17; 95%CI:1.02–1.34) and gonococcal infection (APR = 1.16; 95%CI:1.04–1.28), while in Mali they were sex work duration ≤ 1 year (APR = 1.35; 95%CI:1.10–1.65) and HIV infection (APR = 1.26; 95%CI: 1.06–1.51).

**Conclusion:**

Our study found a very high prevalence of HPV infection as well as high frequency of multiple HPV type infections in FSWs in two countries in West Africa. These findings suggest the necessity to emphasize cervical cancer prevention in this high-risk group.

## Introduction

Cervical cancer is the second most frequent cancer among women and the leading cause of cancer-related death in many countries in Sub-Saharan Africa (SSA) [1]. It is well established that early detection via screening and effective precancer treatment can significantly reduce the incidence of invasive cervical cancer as well as cancer-related death [2, 3]. However, due to limited access to infrastructure, lack of financial and technical resources and well-trained physicians, effective organized cervical cancer screening programs do not exist in many SSA’s countries [4–6]. As a consequence, this part of the world bears the highest burden of cervical cancer in terms of incidence and mortality [7].

Human papillomavirus (HPV) infection is the most common sexually transmitted infection (STI) worldwide [8]. According to 2007 data, the overall prevalence of HPV infection among sexually active women with normal cytology is estimated at 10.4% worldwide. The lowest prevalence rates are observed in Asia (8%) and the highest in Africa (22.1%) [9]. To date, more than 100 HPV genotypes have been identified. According to their oncogenic potential, they are divided into high risk (HR), possible or probable high risk (pHR) and low risk (LR) HPV types [10]. Most of cervical HPV infections are transient and resolve spontaneously. However, persistent infections with HR-HPV types are the etiological agents of cervical pre-malignant and malignant lesions [11]. HPV16 and HPV18 are the most oncogenic genotypes responsible for 70% of all cases of cervical cancer worldwide, while other HPV types like HPV31, HPV33, HPV35, HPV45, HPV52, and HPV58 represent an additional 20% of cervical cancer cases [12].

Benin and Mali are two West African countries where cervical cancer is a major public health issue [13]. Recent data in 2018 reported age-standardized incidence and mortality rates of 23.7 and 20.2 per 100,000 in Benin, while in Mali they were estimated at 43.9 and 36.2 per 100,000, respectively. Thus, in West Africa, Benin and Mali ranked 13^th^ and 3^rd^, respectively, in terms of age-standardized incidence and 12^th^ and 3^rd^ rank according to mortality of cervical cancer [13]. Furthermore, the prevalence of HPV infections in women from the general population with normal cytology is estimated at 26.7% in Benin (2011) [14] and 12% in Mali (2011) [15], but little is known about HPV genotype distribution among these women.

In regions of the world where the HIV epidemic is driven by heterosexual transmission, FSWs and their clients constitute a core group for the spread of HIV/STI to the general population [16]. In addition, because of their high level of sexual activity, FSWs are simultaneously at high risk of contracting HPV infections as well as other STIs, including HIV infection [17]. Like for HIV infection, their clients may spread HPV towards women of the general population after acquiring it from FSWs [18]. A literature review conducted in 2013 reported an overall prevalence of HPV infections of 45.7% (range 2.3% to 100%) among FSWs worldwide [17]. Studies conducted in East and West Africa reported an HPV prevalence of 57.7% in Kenya in 2017 [19]; 26% in Ghana in 2019 [20]; 45.2% in Togo in 2019 [21]; 51.1% in Cote d’Ivoire in 2018 [22]; 66,1% in Burkina Faso in 2006 [23] and 79.8% in Senegal in 2019 [24]. Also, according to the studies conducted in 2017 in Kenya and 2019 in Burkina Faso, high prevalence of multiple HPV type infections has been reported in this group, significantly increasing their risk of cervical pre-cancer and cancer [19, 24]. Understanding determinants of cervical HPV infections in FSWs is essential for developing effective prevention programs. Moreover, with the advent of HPV vaccination programs in African countries, it is essential to better understand the distribution of circulating HPV genotypes in this group. To our knowledge, there is no data published concerning HPV infection among FSWs in Benin and Mali. Therefore, this study aimed to: (1) Estimate HPV prevalence and genotype distribution among FSWs in Mali and Benin as well as the prevalence of multiple HPV type infections in this group, and (2) Identify risk factors associated with HR-HPV infections among FSWs in each country.

## Materials and methods

### Study design and settings

We analyzed baseline data from a prospective cohort study on cervical cancer screening, HPV and HIV infections. From January 2017 to March 2018, the study was conducted in collaboration with three Non-Governmental Organizations (NGOs) in Bamako (Mali) namely SOUTOURA, DANAYA SO and ARCAD-SIDA. These NGOs are the most active in the field of STI/HIV prevention in Mali, with a recognized leadership and trusted by FSWs. The details of NGOs from Mali are shown in a previous publication [25]. In Benin, we collaborated with “Santé et Développement (SED)”, a NGO working with FSWs for over 20 years with confirmed proficiency in STI/HIV prevention and “SOLIDARITÉ”, the local association of women practicing sex work, which operates a network of peer-educators (PEs).

### Study planning, recruitment procedures and study population

For uniform procedures in both countries, we have carefully planned our study. Before starting the activities, we organized a training session targeting physicians at the “Dispensaire des IST (DIST)” in Cotonou and at the ARCAD-SIDA STI clinic in Bamako. These sessions covered theorical and practical aspects of cervical cancer screening, review of questionnaire and practical issues related to data collection procedures in the field.

For field work issues, we recruited four PEs from each participating NGO in Mali, two PEs as well as two facilitators in Benin. Specific training was provided to these PEs and facilitators to familiarize them with the survey procedures. A cervical cancer screening awareness campaign preceded the recruitment of FSWs in the two cities. These activities were performed by trained PEs and senior staff from the participating NGOs in the bars, brothels, hotels, etc. Women interested to participate in the study were invited to come to the DIST in Cotonou (STI clinic offering adapted services to FSWs) or to the ARCAD-SIDA STI clinic specialized in FSW care in Bamako. The service package offered in these two FSW-friendly STI clinics includes small talk discussion for behavior change, peer education, condom use demonstration, as well as free distribution of condoms and lubricants. In addition, they provide STI care using syndromic management as well as HIV testing and treatment for free of charge.

### Inclusion and exclusion criteria

A FSW was defined as any woman who receives money or gifts in exchange for sex. To be included in the study participants had to fulfill the three criteria as follows: (a) being a FSW in the study settings for at least 6 months; (b) being referred by one of the PEs of the participating NGOs; and (c) being aged between 18 and 65 years old. All FSWs who had previously been diagnosed with cervical cancer, who had a hysterectomy and those who were pregnant were excluded from the study.

### Data collection

At the clinics, the study procedures were explained to each FSW and informed consent was obtained. A questionnaire was administered face-to-face by qualified interviewers to collect data on socio-demographic characteristics, including age, marital status, educational level, country of origin; behavioral and sex work characteristics like the frequency of alcohol consumption, drug consumption, number of cigarette packs smoked per week, age at first sexual intercourse, total number of sexual partners in the last week of work, number of paying clients in the last week of work, having regular partner, frequency of condom use, duration of sex work, place where practicing sex work, oral and anal sexual intercourse, vaginal douching; reproductive health variables as well as family and medical histories, such as parity, number of abortions, number of vaginal and cesarian deliveries, history of cervical cancer, ovarian cancer, vaginal cancer or breast cancer, family history of these cancers; history of STIs in the last six months.

Following the interview, each woman underwent a gynecological examination performed by a well-trained physician. Vaginal and cervical swabs were obtained from each participant for curable STI testing. A cytobrush (Rovers Medical Devices B.V. Oss, The Netherlands) was used to collect cells from the endocervix and ectocervix and stored in tubes containing 3 ml of fresh PBS (phosphate-buffered saline) for HPV DNA testing. Cervical cancer screening was performed using visual inspection methods (VIA/VILI). Finally, a venous blood sample was taken for HIV testing and CD4 count.

### Laboratory analyses

In both countries, the same procedures were used for the following tests. Wet mounts of the vaginal swabs were microscopically examined immediately for *Trichomonas vaginalis* and *Candida Albicans*. The diagnosis of bacterial vaginosis (BV) was made using Nugent score.

For *Neisseria gonorrhoeae* and *Chlamydia trachomatis* detection, we used different tests. In Benin, these infections were detected using the NG/CT Probetec® assay from Becton Dickenson (Cockeysville, MD, USA), while in Mali, these infections were detected using the Abbott Real-Time CT/NG assay. These assays were carried out according to the manufacturers’ instructions. These two tests have similar sensitivity and specificity as reported elsewhere [26, 27]. All FSWs with laboratory diagnosed STIs received appropriate treatment, free of charge.

Both, Mali and Benin have the same national HIV testing algorithms and we thus used the same tests for the detection of HIV antibodies for all the study participants. Alere Determine HIV-1/2 test (Alere Medical Co. Ltd) was used as first line and positive specimens were then confirmed with a rapid and discriminatory test SD Bioline (Giheung-gu, Yongin-si, Korea). Women testing positive for HIV were immediately offered antiretroviral therapy.

### Amplification and detection of HPV DNA

HPV testing was performed as part of the study. In both countries, endocervical and ectocervical cells were centrifuged at 3000 rpm for 5 minutes at 4°C for cervical cell concentration. Cell pellets were stored at -20°C until DNA extraction. At the end of the study, all cell pellets were sent to Montreal (Canada) in a laboratory specialized in HPV testing. HPV Genotyping was performed using the Linear Array HPV genotyping test (Roche Molecular Systems, Inc., Laval, Qc, Canada). This test identifies 36 genotypes by hybridization on a linear array of PGMY-generated amplicons with 34 type-specific probes for HPV-6, -11, -16, -18, -26, -31, -33, -35, -39, -40, -42, -45, -51, -53, -54, -55, -56, -58, -59, -61, -62, -64, -66, -67, -68, -69, -70, -71, -72, -73, -81, -83, -84, and -89, two probes for two variants of HPV-82, and one probe that cross-reacts with HPV-33, -35, -52, and -58. Amplification and type detection were performed according to the manufacturer’s recommendations [28]. The cross-reactivity between HPV 52 and HPV-33, 35 or 58 was further tested with a Real-Time PCR assay specific for HPV52 as described elsewhere [29].

### Outcome variable

The dependent variable was “HR-HPV” defined as being positive for at least one HR-HPV type.

### Statistical analysis

Data were analyzed using SAS version 9.4 (SAS institute, Inc, Cary, North Carolina, USA). We used descriptive statistics to analyze demographic, behavioral and sex work characteristics. The results were presented as percentages, means with standard deviations (± SD) or medians with inter-quartile ranges (IQR). Pearson’s Chi-Square test or Fisher’s exact test were used for categorical variables, the Student’s *t* test for continuous variables and rank test with median score. We also computed descriptive statistics to estimate HPV prevalence rates. An exact binomial 95% confidence interval (95%CI) was calculated for each prevalence rate. To identify factors associated with HR-HPV infection, we carried out, for each country, univariate and multivariate log-binomial regression models with a robust “sandwich” variance estimator to calculate the adjusted prevalence ratios (APRs) with 95% CI. All variables significant at p ≤ 0.2 (as recommended for variable selection [30]) in univariate analysis or known from the literature as potential risk factors were computed into multivariate log-binomial regression models to build the full model. Manual backwards elimination procedures were applied to remove covariates from the full model if they were neither significant (p-value ≥ 0.05) or changing the effect estimate for the association of HR-HPV and other variables by more than 10%.

### Ethical issues

The study was reviewed and approved by the ethics committees of the School of medicine of Bamako, Mali (#n^o^2017/93/CE/FMPOS), and the “*CHU de Québec-Université Laval”* (#2017–3313), as well as the National Ethics Committee for Health Research in Benin (# n^o^01 du 25 janvier 2017). The objectives, procedures and potential risks related to the study were explained to each woman and written informed consent was obtained before enrolment. Consenting participants signed or apposed their fingerprint on the consent forms. In Mali, participants received 5000 CFA (about US$8.40), while in Benin, they received 3000 CFA (about US$5) for compensation of transportation and the time spent at the clinic. The amount of compensation was slightly different between the two countries because of the socio-economic context (the cost of living is slightly higher in Mali than in Benin). Condoms and lubricants were distributed for free to each woman.

## Results

### Socio-demographic and sexual behavioral characteristics

A total of 710 FSWs were approached (337 in Benin and 373 in Mali). Of these 20 FSWs were excluded in Mali (one previous cervical cancer and 19 pregnancies) and 25 FSWs in Benin (9 had their menstruations and 16 pregnancies). A total of 665 women were thus included in the study, 312 in Benin and 353 in Mali. FSWs from Benin were older than those from Mali, the mean (± SD) age was 35.0 ± 10.7 versus 26.8 ± 7.6 years respectively (p < .0001; Table 1). FSWs in Mali were mostly never married (69%), whereas they were mostly separated/widowed/divorced in Benin (55.8%). In both countries nearly 40% were uneducated. Mean age at first sexual intercourse and mean age at first paid sex were lower in Mali (p < .0001). Vaginal douching was more frequently practiced in Benin (79.2%) as compared with Mali FSWs (40.3%, p < .0001). Curable STIs (*C*. *trachomatis*, *N*. *gonorrhoeae*, *T*. *vaginalis*) were more common in Mali than in Benin (p < 0.0001). HIV prevalence was 26.3% in Benin versus 20.4% in Mali and the proportions of positive VIA/VILI tests were 20.2% and 10.5%, respectively.

**Table 1:** Sociodemographic, reproductive, sex works and biological characteristics among female sex workers in Cotonou (Benin) and Bamako (Mali).

Characteristic	Benin	Mali	p-Value^¶^
	N = 312	N = 353	
	n (%)	n (%)	
**Sociodemographic Characteristics**			
Age in years, mean (± SD)	35.0 (10.7)	26.8 (7.6)	< .0001^£^
Age in years			< .0001
18–24	62 (19.9)	161 (45.6)	
25–29	56 (18.0)	87 (24.6)	
30–34	41 (13.1)	51 (14.4)	
35–39	40 (12.8)	27 (7.7)	
≥ 40	113 (36.2)	27 (7.7)	
Education level			0.830
Uneducated	120 (38.6)	140 (39.7)	
Primary	127 (40.8)	147 (41.6)	
Secondary or higher	64 (20.6)	66 (18.7)	
Marital status			< .0001
Married	28 (8.9)	27 (7.7)	
Separated /widowed/divorced	174 (55.8)	82 (23.2)	
Never married	110 (35.3)	244 (69.1)	
Country of origin			< .0001
Benin	143 (45.8)	3 (0.9)	
Nigeria	91 (29.2)	28 (7.9)	
Mali		262 (74.2)	
Ghana	21 (6.7)	4 (1.1)	
Others*	57 (18.3).	56 (15.9)	
**Reproductive Characteristics**			
Number of children			< .0001
0	55 (17.7)	110 (31.2)	
1	77 (24.8)	115 (32.6)	
2	61 (19.6)	71 (20.1)	
3	51 (16.4)	29 (8.2)	
≥ 4	67 (21.5)	28 (7.9)	
**Behavioral and Sex Work Characteristics**			
Alcohol consumption			< .0001
Ever	226 (73.1)	159 (45.0)	
Never	83 (26.9)	194 (55.0)	
Drug abuse			0.483
Ever	21 (6.8)	29 (8.2)	
Never	289 (93.2)	324 (91.8)	
Tobacco			0.000
Never	251 (81.2)	243 (68.8)	
Less than 10 cigarettes a week	36 (11.7)	53 (15.0)	
Ten cigarettes and more a week	22 (7.1)	57 (16.2)	
Place of work			< .0001
Bar-based^#^	141 (45.6)	318 (90.0)	
Home-based	80 (25.9)	20 (5.7)	
Others^$^	88 (28.5)	15 (4.3)	
Age at first sexual intercourse; mean (SD)	17.5 (2.7)	15.3 (2.9)	< .0001^£^
Age at first paid sex; mean (SD)	27.2 (9.1)	21.6 (7.0)	< .0001^£^
Duration of sex work in years, median (IQR)	5 (2–10)	4 (2–7)	0.029^©^
Latest week total number of sexual partners^&^; median (IQR)	12 (6–20)	10 (5–20)	0.000^©^
Number of paying clients, last 7 days of work; median (IQR)	12 (6–20)	10 (4–20)	0.000^©^
Having a regular sexual partner (boyfriend or husband)	173 (55.5)	239 (67.7)	0.001
Always used condom with paying clients (last 7 days of work)	281 (90.1)	336 (95.7)	0.004
**Intravaginal practice**			
Used vaginal douching before and after sex	247 (79.2)	137 (40.3)	< .0001
Insert product into vagina	17 (5.5)	33 (9.4)	0.059
**Biological Characteristics**			
HIV	82 (26.3)	72 (20.4)	0.073
*N*. *gonorrhoeae*	43 (13.8)	85 (24.2)	0.001
*C*. *trachomatis*	23 (7.4)	49 (14.0)	0.007
*T*. *vaginalis*	5 (1.6)	13 (3.7)	0.099
Bacterial vaginosis (Nugent score ≥ 7)	164 (52.6)	83 (23.5)	< .0001
*C*. *albicans*	6 (1.9)	43 (12.2)	< .0001
**VIA/VILI positive**	60 (20.2)	37 (10.5)	0.001

Abbreviations: SD, Standard Deviation; IQR, interquartile range; HIV, Human Immunodeficiency Virus.

*Burkina Faso, Togo, Ghana, Côte d’Ivoire, Guinea, Senegal, Mauritania.

^#^ Bars, Hotel, Nightclub

^$^Private home, street.

^&^Included all sexual partners.

^¶^ p-Value from Pearson Chi-Square.

^£^p-Value from Student’s *t* test.

^©^p-Value from rank test using score of median.

### HPV prevalence and type-specific distribution

Among 665 women enrolled, HPV data were not available for 6 FSWs, three in Mali and three in Benin, because of invalid cervical samples, but all other variables were available for these FSWs. The prevalence rates of any HPV among FSWs were 95.5% (95%CI: 92.5–97.5) and 81.4% (95%CI: 77.0–85.4) in Benin and Mali, respectively (Table 2). HR-HPV prevalence was higher among FSWs from Benin than those from Mali (87.1% versus 62.3%; p < .0001). A similar pattern was found with LR-HPV types (77.4% versus 55.4%, respectively; p < .0001). HPV prevalence was 87.5% in HIV-positive FSWs vs 86.9% among HIV-negative FSWs in Benin. These statistics in Mali were 71.8% and 59.9% for HIV-positive and HIV-negative FSWs, respectively.

**Table 2:** HPV Genotype distribution among female sex workers in Cotonou (Benin) and Bamako (Mali).

	Benin			Mali			p-Value^$^
	N* = 312			N* = 353			
	n	%	95%CI	n	%	95%CI	
**Any HPV**	295	95.5	92.5–97.5	285	81.4	77.0–85.4	< .0001
**HR-HPV**							
HR-HPV	269	87.1	82.3–90.6	218	62.3	57.0–67.4	< .0001
HPV16	113	**36.6**	**31.2–42.2**	55	**15.7**	**12.1–20.0**	
HPV18	64	**20.7**	**15.3–25.7**	34	9.7	6.8–13.3	
HPV31	16	5.2	3.0–8.3	23	6.6	4.2–9.7	
HPV33	34	11.0	7.5–14.5	22	6.3	4.0–9.4	
HPV35	72	**23.2**	**18.7–28.4**	43	**12.3**	**9.0–16.2**	
HPV39	10	3.2	1.6–5.9	27	7.7	5.2–11.0	
HPV45	47	15.2	11.4–19.7	31	8.9	6.1–12.3	
HPV51	20	6.5	4.0–9.2	50	**14.3**	**10.79–18.4**	
HPV52	89	**28.8**	**23.8–34.2**	45	**12.9**	**9.5–16.8**	
HPV56	13	4.2	2.3–7.1	17	4.9	2.9–7.7	
HPV58	116	**37.5**	**32.1–43.2**	41	**11.7**	**8.5–15.6**	
HPV59	28	9.1	6.1–12.8	38	10.9	7.8–14.6	
**Probable HR-HPV**							
pHR-HPV	153	49.5	43.8–55.2	150	42.9	37.6–48.3	0.087
HPV26	4	1.3	0.3–3.3	10	2.9	1.4–5.2	
HPV34	1	0.3	0.0–1.8	1	0.3	0.0–1.6	
HPV53	33	**10.7**	**7.5–14.7**	40	**11.4**	**8.3–15.2**	
HPV66	34	**11.0**	**7.7–15.0**	35	**10.0**	**7.1–13.6**	
HPV67	13	4.2	2.3–7.1	10	2.9	1.4–5.2	
HPV68	68	**22.0**	**17.5–27.1**	45	**12.9**	**9.5–16.8**	
HPV70	26	**8.4**	**5.6–12.1**	15	4.3	2.4–7.0	
HPV73	17	5.5	3.2–8.7	22	**6.3**	**4.0–9.4**	
HPV82	18	**5.8**	**3.5–9.1**	29	**8.3**	**5.6–11.7**	
**LR-HPV**							
LR-HPV	239	77.4	72.3–81.9	194	55.4	50.1–60.7	< .0001
HPV6	17	5.5	3.2–8.7	29	8.3	5.6–11.7	
HPV11	17	5.5	3.2–8.7	5	1.4	0.4–3.3	
HPV40	8	2.6	1.1–5.0	10	2.9	1.4–5.2	
HPV42	37	**12.0**	**8.6–16.1**	24	**6.9**	**4.4–10.0**	
HPV44	18	5.8	3.5–9.1	18	5.1	3.1–8.0	
HPV54	18	5.8	3.5–9.1	24	**6.9**	**4.4–10.0**	
HPV61	71	**23.0**	**18.4–28.1**	45	**12.9**	**9.5–16.8**	
HPV62	110	**35.6**	**30.3–41.2**	53	**15.1**	**11.6–19.3**	
HPV69	11	3.6	1.8–6.3	5	1.4	0.5–3.3	
HPV71	11	3.6	1.8–6.3	5	1.4	0.4–3.3	
HPV72	47	15.2	11.4–19.7	13	3.7	2.0–6.3	
HPV81	73	**23.6**	**19.0–28.8**	34	**9.7**	**6.8–13.3**	
HPV83	45	14.6	10.8–19.0	30	8.6	5.9–12.0	
HPV84	50	**16.2**	**12.3–20.8**	37	**10.6**	**7.6–14.3**	
HPV89	31	10.0	6.9–13.9	29	8.3	5.6–11.7	

Abbreviations: HPV, Human papillomavirus; HR-HPV, High-risk human papillomavirus; pHR-HPV, probable high-risk human papillomavirus; LR-HPV, Low-risk human papillomavirus; CI, 95% Confidence Interval.

Any HPV was defined as being positive for at least one of the 36 HPV types detected

HR-HPV was defined as being positive for at least one HR-HPV type

pHR-HPV was defined as being positive for at least one pHR-HPV type

LR-HPV was defined as being positive for at least one LR-HPV type

Numbers in bold represent the top five HPV genotypes for each country.

^$^p-Value calculated with Pearson’s χ2 test or Fisher’s exact test to compare frequencies between countries for Any HPV, Any HR-HPV, Any pHR-HPV and Any LR-HPV.

*For each country, HPV data were not available for three female sex workers.

HPV genotype distribution varied widely between the two countries with HPV58 (37.5%), HPV16 (36.6%) and HPV52 (28.8%) being the top three in Benin, compared to HPV16 (15.7%), HPV51 (14.3%) and HPV52 (12.9%) in Mali. Concerning LR-HPV the top three in Benin were: HPV62 (35.6%), HPV81 (23.6%) and HPV61 (23.0%). This profile in Mali was HPV62 (15.1%), HPV61 (12.9%) and HPV84 (10.6%).

The prevalence of HR-HPV multiple type infections (≥ 2 HR-HPV) among FSWs was high in this study (Table 3). Multiple HR-HPV type infections occurred in 61.8% and 35.4% of FSWs in Benin and Mali respectively (p < 0.0001). A similar profile was observed for LR-HPV types.

**Table 3:** Multiple HPV infections detected among female sex workers in Cotonou (Benin) and Bamako (Mali).

	Benin			Mali			p-Value^$^
	N* = 312			N = 353			
	n	%	95%CI	n	%	95%CI	
**Number of types detected any type**							
0 Type	14	4.5	2.5–7.5	65	18.6	14.6–23.1	
1 Type	24	7.8	5.4–11.4	63	18.0	14.1–22.4	
2 Types	35	**11.3**	**8.0–15.4**	59	**16.9**	**13.1–21.2**	
3 Types	39	**12.6**	**9.1–16.9**	54	**15.4**	**11.8–19.7**	
4 Types	45	**14.6**	**10.8–19.0**	32	9.1	6.3–12.7	
5 Types	47	**15.2**	**11.4–19.7**	23	6.6	4.2–9.7	
6 Types	44	**14.2**	**10.5–18.6**	22	6.3	3.7–8.8	
7 Types	25	8.1	5.3–11.7	11	3.1	1.3–5.0	
≥ 8 Types	36	**11.6**	**8.3–15.8**	21	6.0	3.5–8.5	
**Multiple infection with any type**							
≥ 2 Types	271	87.7	83.5–91.2	222	63.4	58.1–68.5	< .0001
**Number of HR-HPV type detected**							
0 Type HR-HPV	40	12.9	9.4–17.2	132	37.7	32.6–43.0	
1 Type HR-HPV	78	25.2	20.5–30.5	94	26.9	22.3–31.8	
2 Types HR-HPV	85	**27.5**	**22.6–32.9**	70	**20.0**	**15.9–24.6**	
3 Types HR-HPV	64	**20.7**	**16.3–25.7**	32	9.1	6.3–12.6	
4 Types HR-HPV	31	**10.0**	**6.9–13.9**	16	4.6	2.6–7.3	
5 Types HR-HPV	8	2.6	1.1–5.0	4	1.1	0.3–2.9	
6 Types HR-HPV	3	1.0	0.2–2.8	2	0.6	0.1–2.0	
**Multiple infection with HR-HPV types**							
≥ 2 Types HR-HPV	191	61.8	56.1–67.3	124	35.4	30.4–40.7	< .0001
**Number of pHR-HPV type detected**							
0 Type pHR-HPV	156	50.5	44.7–56.2	200	57.1	51.8–62.4	
1 Type pHR-HPV	105	34.0	28.7–39.6	107	30.6	25.8–35.7	
2 Types pHR-HPV	37	**12.0**	**8.6–16.1**	32	9.1	6.3–12.7	
3 Types pHR-HPV	9	2.9	1.3–5.5	8	2.3	1.0–4.5	
4 Types pHR-HPV	2	0.6	0.1–1.5	3	0.9	0.2–2.5	
**Multiple infection with pHR-HPV types**							
≥ 2 Types pHR-HPV	48	15.5	11.7–20.1	43	12.3	9.0–16.2	0.228
**Number of LR-HPV type detected**							
0 Type LR-HPV	70	22.7	18.1–27.7	156	44.6	39.3–50.0	
1 Type LR-HPV	75	24.3	19.6–29.5	104	29.7	25.0–34.8	
2 Types LR-HPV	72	**23.3**	**18.7–28.4**	48	**13.7**	**10.3–17.8**	
3 Types LR-HPV	47	**15.2**	**11.4–19.7**	20	5.7	3.5–8.7	
4 Types LR-HPV	28	9.1	6.1–12.8	13	3.7	2.0–6.3	
5 Types LR-HPV	12	3.9	2.0–6.7	5	1.4	0.5–3.3	
6 Types LR-HPV	3	1.0	0.2–2.8	4	1.1	0.3–2.9	
7 Types LR-HPV	2	0.7	0.1–2.3	-	-	-	
**Multiple infection with LR-HPV types**							
≥ 2 Types LR-HPV	164	53.7	47.3–58.8	90	25.7	21.2–0.6	< .0001
**Prophylactic vaccine type**							
Any 4-valent vaccine types	175	65.1	59.0–70.7	94	43.1	36.5–50.0	< .0001
0 type	94	34.9	29.3–50.0	124	56.9	50.0–63.5	
1 type	145	53.9	47.8–60.0	71	32.6	26.4–39.2	
2 types	28	**10.41**	**7.0–14.7**	20	9.2	5.7–13.8	
3 types	2	0.7	0.0–2.7	3	1.4	0.2–4.0	
Any 9-valent vaccine types	246	91.5	88.1–94.9	167	76.6	70.4–82.1	< .0001
0 Type	23	8.6	5.5–12.6	51	23.4	17.4–29.6	
1 Type	89	33.1	27.5–39.1	91	41.7	35.1–48.8	
2 Types	75	**27.9**	**22.6–33.7**	50	**22.9**	**17.5–29.1**	
3 Types	60	**22.3**	**17.5–27.8**	15	6.7	23.9–11.1	
4 Types	20	7.4	4.6–11.3	9	4.1	1.9–7.7	
5 Types	2	0.7	0.0–2.6	2	0.9	0.1–3.2	

Abbreviations: HPV, Human papillomavirus; HR-HPV, High-risk human papillomavirus; pHR-HPV, probable high-risk human papillomavirus; LR-HPV, Low-risk human papillomavirus; CI, 95% Confidence Interval.

Multiple infection with any HPV was defined as being positive for two or more HPV types.

Multiple infection with HR-HPV was defined as being positive for two or more HR-HPV types.

Multiple infection with pHR-HPV was defined as being positive for two or more pHR-HPV types.

Multiple infection with LR-HPV was defined as being positive for two or more LR-HPV types.

Any 4-valent = HPV6 or HPV11 or HPV16 or HPV18.

Any 9-valent = HPV6 or HPV11 or HPV16 or HPV18 or HPV31 or HPV33 or HPV45 or HPV52 or HPV58.

Numbers in bold represent multiple HPV type infections with a prevalence rate ≥ 10%.

^$^p-Value calculated with Pearson’s χ2 test or Fisher’s exact test to compare frequencies between countries for ≥ 2 HPV types, ≥ 2 HR-HPV, ≥ 2 pHR-HPV and ≥ 2 LR-HPV.

*For each country, HPV data were not available for three female sex workers.

To evaluate the potential effectiveness of the HPV vaccines in FSWs, we estimated the overall prevalence rate and multiple infections with HPV types covered by available vaccines (Table 3). This analysis was restricted to HR-HPV positive FSWs. In Benin, about 65.1% of HR-HPV positive FSWs had at least one of the 4 genotypes covered by the Gardasil-4 vaccine versus 43.1% in Mali (p < 0.0001). Regarding the Gardasil-9 vaccine, 91.2% and 76.6% of HR-HPV infected FSWs harbored at least one HPV type prevented by the 9-valent HPV vaccine in Benin and Mali, respectively (p < 0.0001). Finally, none of the study participants had previously received any HPV vaccine at the time of the study.

### Risk factors for HR-HPV infections

The country-specific risk factors for HR-HPV types are shown in Table 4. In Benin, the main risk factors for HR-HPV infections were age < 25 years (APR = 1.25; 95%CI: 1.06–1.47), p-trend test = 0.015, vaginal douching before and after sex (APR = 1.17; 95%CI: 1.02–1.34) and gonorrhea infection (APR = 1.16; 95%CI: 1.04–1.28). In Mali, these figures were being home-based FSWs (APR = 2.13; 95%CI: 1.17–3.86), sex work duration ≤ 1 year (APR = 1.35 95%CI: 1.10–1.65) and being HIV positive (APR = 1.26 95%CI: 1.06–1.51). There was an inverse association between high number of clients (≥ 5) compared to a lower number (< 5; p < 0.001).

**Table 4:** Risk factors associated with HR-HPV infection among female sex workers in Cotonou (Benin) and Bamako (Mali).

	Benin				Mali			
Characteristics	n/N	%HR-HPV	APR [95%CI	p-Value^£^	n/N	%HR-HPV	APR [95%CI	p-Value^£^
**Age in years**			** **	**0.063 **				0.489
18–24	59/62	95.2	**1.25 [1.06–1.47]**		108/161	67.1	1.27 [0.81–1.96]	
25–29	51/56	91.1	1.15 [1.00–1.33]		49/86	57.0	1.07 [0.68–1.67]	
30–34	34/40	85.0	1.05 [0.89–1.23]		30/51	58.8	1.06 [0.67–1.69]	
35–39	34/40	85.0	1.06 [0.90–1.24]		18/25	72.0	1.24 [0.76–2.00]	
≥ 40	91/111	82.0	1.00		13/25	48.2	1.00	
**Trend p-value**				**0.015**				0.320
**Education level**				0.239				0.042
Uneducated	104/119	87.4	0.97 [0.87–1.07]		78/139	56.1	0.83 [0.64–1.08]	
Primary	106/126	84.1	0.92 [0.82–1.02]		99/145	68.3	1.05 [0.83–1.34]	
Secondary or higher	58/63	92.1	1.00	-	41/66	62.1	1.00	
**Having a regular sexual partner (boyfriend or husband)**				0.947				0.920
Yes	151/171	88.3	1.00		139/237	62.9	1.00	
No	118/138	85.5	1.00 [0.90–1.10]		69/113	61.1	0.99 [0.81–1.20]	
**Age at first sexual intercourse**				0.352				0.238
< 18	105/122	86.1	0.94 [0.85–1.04]		164/258	63.6	1.18 [0.93–1.50]	
≥18	119/134	88.8	1.00		39/71	54.5	1.00	
Unknown	45/53	84.9	0.93 [0.82–1.06]		15/21	71.4	1.36 [0.93–2.00]	
**Place of work**				0.165				**0.021**
Bar-based^#^	118/139	84.9	0.91 [0.82–1.01]		196/315	62.2	1.63 [0.93–2.83]	
Home-based	70/79	88.6	0.99 [0.89–1.11]		16/20	80.0	**2.13 [1.17–3.86]**	
Others^$^	79/88	89.8	1.00		6/15	40.0	1.00	
**Duration of sex work in years**				0.350				**0.021**
≤ 1	40/47	91.5	1.00 [0.90–1.12]		43/57	75.4	**1.35 [1.10–1.65]**	
2–3	54/62	87.1	0.95 [0.84–1.08]		65/107	60.8	1.03 [0.85–1.26]	
≥ 4	133/156	85.3	1.00		105/179	58.7	1.00	
Unknown	39/44	88.6	1.08 [0.97–1.20]		5/7	71.5	1.03 [0.65–1.64]	
**Number of clients in the last seven days**				0.509				**<0.001**
<5	48/57	84.2	1.00		66/91	72.5	1.00	
5–14	109/122	89.0	1.07 [0.95–1.21]		72/133	54.1	**0.69 [0.57–0.84]**	
≥ 15	112/130	86.2	1.06 [0.94–1.19		78/124	62.9	**0.80 [0.67–0.96]**	
**Always used condom with paying clients (last 7 days of work)**				0.189				0.314
Yes	244/278	87.8	1.11 [0.95–1.30]		207333	62.2	0.84 [0.59–1.18]	
No	25/31	80.7	1.00		10/15	66.7	1.00	
**Used vaginal douching before and after sex**				**0.021**				0.491
Yes	219/245	89.4	**1.17 [1.02–1.34]**		90/135	66.7	1.06 [0.90–1.26]	
No	50/64	78.1	1.00		122/202	60.4	1.00	
**HIV**				0.236				**0.010**
Yes	70/80	87.5	1.07 [0.96–1.19]		51/71	71.8	**1.26 [1.06–1.51]**	
No	199/229	86.9	1.00		167/279	59.9	1.00	
***N*. *gonorrhoeae***				**0.006**				0.618
Yes	40/43	93.2	**1.16 [1.04–1.28]**		57/85	67.1	1.05 [0.87–1.26]	
No	229/266	86.1	1.00		159/263	60.5	1.00	
***C*. *trachomatis***				0.320				0.234
Yes	19/22	86.4	0.92 [0.79–1.08]		30/48	62.5	0.87 [0.69–1.09]	
No	250/287	87.1	1.00		186/300	62.0	1.00	
***T*. *vaginalis***				0.181				0.886
Yes	2/5	40.0	0.57 [0.25–1.30]		6/11	54.6	0.96 [0.58–1.60]	
No	267/304	87.8	1.00		212/339	62.4	1.00	
**Bacterial vaginosis**				0.332				0.177
Nugent score < 7	146/164	89.0	1.00		57/81	70.4	1.13 [0.95–1.36]	
Nugent score ≥ 7	123/145	84.8	1.05 [0.95–1.15]		161/269	59.9	1.00	
***C*. *Albicans***				0.777				0.060
Yes	5/6	83.3	0.97 [0.78–1.20]		32/42	76.2	1.21 [0.99–1.47]	
No	264/303	84.8	1.00		186/308	60.4	1.00	

Abbreviations: HR-HPV, High-Risk Human papillomavirus; HIV, Human Immunodeficiency Virus; CI, 95% Confidence Interval.

Bolded results represent those that are statistically significant.

^#^Bars, Hotel, Nightclub

^$^Private home, street.

n = numerator, positive cases. N = denominator, total of each category

^£^p-Value from Wald Statistics for Type 3 GEE Analysis.

## Discussion

This is the first epidemiological study on HPV infection among FSWs in Benin and Mali. Our findings show a high overall prevalence rate of HPV infection as well as a high rate of HR-HPV types among this group in both countries. Similarly, there was a high rate of multiple HPV type infections among them. We also noted a wide variation of HPV genotype distribution among FSWs between both countries. Furthermore, we identified several factors associated HR-HPV infections that were different between countries.

Prevalence rates of HPV infection of 95.5% in Benin and 81.4% in Mali were several-fold higher than those found among women in the general population (26.7% in Benin and 12% in Mali according to 2011 data) [14, 15]. These results corroborate previous reports [31] and are partly explained by the cumulative exposure of FSWs to high number of sexual partners and other STIs.

Furthermore, compared with other studies among FSWs in West Africa where HR-HPV prevalence rates varied from 32.9% - 72.5% [21, 24], the prevalence rate of HR-HPV infections was higher in our study. There was also a high prevalence of multiple infections (≥ 2 HR-HPV) as reported elsewhere among FSWs in SSA settings [17, 19, 24]. Two hypotheses may explain these differences. First, HIV prevalence among FSWs participating in other HPV studies in West Africa (10.6% to 15.4) [21, 24] is lower than what we found (26.3% among FSWs in Benin and 20.4% in Mali). The strong association between HIV and HPV is well documented and a higher HIV prevalence could thus lead to a higher HPV prevalence [32]. Secondly, the HPV genotyping methods and the number of HPV types detected differ between studies cited here (21 HPV to 28 HPV) [21, 24]. We used a more sensitive assay, detecting up to 36 HPV types.

A striking feature of HPV infection in FSWs is the wide variation of genotypes across countries. HPV58 and HPV16 were the most frequent in Benin, while in Mali, the predominant genotypes were HPV16 and HPV51. These two prevalent genotypes are quite different from those observed in Madagascar [33], but shared the presence of one HR-HPV (HPV16) with observations in Ghana, Senegal and Kenya [19, 20, 24]. Such findings confirm the epidemiological particularity of circulating HPV types in Sub-Saharan Africa [9].

Like elsewhere [20, 21], in Benin, the main potential risk factors of HR-HPV were younger age, gonorrhea as well as vaginal douching. Multiple sexual partners and immature cervix producing inadequate cervical mucus are known factors increasing the likelihood of acquiring HPV infection in young women [34, 35]. Similarly, the associations between HR-HPV and could partly be explained by risky behavior or because of the chronic inflammation caused by this infection, which would facilitate the acquisition of HPV [36, 37]. On the other hand, the relationship between vaginal douching and higher rates of genital infections, including STIs/HPV, is well documented in the general population [38, 39]. Supporting these findings are local immune system disturbance and removal of the cervical mucus protective barrier [40]. In contrast, a study in Cambodia found less HPV infections in FSWs practicing vaginal douching just after sexual intercourse [41]. Such controversies, combined with the rarity of studies exploring the link between HPV infection and vaginal douching among FSWs, despite its common practice among this population in SSA [42], suggest the need for additional studies.

In Mali the highest prevalence of HR-HPV was observed in home-based FSWs. While the relation between HIV infection and sex work place is well documented [43], little is known about the link between HPV infection and sex work place. Differences in number and social categories of sexual partners, prevalence of other STIs, cumulative exposure according to place of sex work could potentially explain our findings. As reported elsewhere [44], shorter sex work duration association with increased HPV prevalence shares a mechanism similar to that of younger age. Our analysis revealed a moderate association between HIV and HR-HPV infections. Supporting this finding is the HIV-induced immunosuppression that can increase the susceptibility to virus acquisition as well as the inability to eliminate HPV infection [45, 46]. Unexpectedly, we noted an inverse association between the high number of sexual partners and HPV infections. This is probably due to the high HIV prevalence among FSWs with < 5 clients as observed in our data publish elsewhere [25].

### Limitations

We studied the epidemiology of HPV infections among FSWs from a convenience sample. To minimize selection bias, we used different recruitment approaches either by the PEs or FSW leaders. In addition, self-reported information on risky behaviors such as number of sexual partners, age at first sexual interaction, etc., may be subject to recall and social desirability bias. Indeed, due to stigma or social desirability, this information collected may be underestimated. However, such misclassification bias would be limited in our study since the data were collected by trusted qualified interviewers. Failure to measure certain variables could cause residual confounding in our analyses on risk factors. To deal with this bias, we adjusted for a large number of potential confounding variables based on the literature. Furthermore, the associations we found between several factors and HR-HPV cannot be viewed as causal because of the cross-sectional nature of the data. In addition, although our results reflect current HPV infection status, the lack of information about HPV antibodies in the serum, which is reported to be a marker of past infections [47], constitute another limitation of this study. Finally, our results may not be generalizable to other FSW populations in West Africa because of the different characteristics observed in FSWs.

### Implications

Understanding the distribution of HPV genotypes among FSWs is crucial to estimating the burden of HPV infections as well as the future impact of HPV vaccines within this high-risk group. The high prevalence of HR-HPV and other STIs/HIV found among FSWs implies an increased risk of cervical cancer.

Currently, the bivalent and quadrivalent vaccines are approved in both countries, but they are not integrated in the routine immunization program. However, these vaccines are available in private pharmacies. The relative low representativeness of HPV types covered by the quadrivalent vaccine like HPV6, HPV11 and HPV18 may question the potential effectiveness of this vaccine in this group. The presence of HPV16, HPV52 and HPV58 in the top three would favor the nonavalent vaccine for cervical cancer prevention. Since evidences support vaccination against HPV in sexually active women up to the age of 44 years [48], FSW immunization program may be one of the most promising strategies to decrease the HPV burden. However, socio-cultural and financial barriers in most SSA countries may impact the implementation and the effectiveness of programs, at least on the short term. The high rates of positive VIA/VILI found in our study call for an emphasis on secondary prevention, namely cervical cancer screening using HPV molecular testing [3]. An integration of these services to the package of services, as recommended by international professional societies, would be a valuable strategy.

## Conclusion

Our study, the first in Benin and Mali, showed a high prevalence of HR-HPV infections among FSWs. These results make FSW a priority group for cervical cancer prevention programs adapted to their context. Additionally, the identification of the predominant HPV genotypes in this population shed the light on the vaccine potentially needed in this specific group.

## Supporting information

S1 File (ZIP)
