# Supplementary material for: Human papillomavirus genotype distribution and factors associated among female sex workers in West Africa
Source: PLoS One. 2020 Nov 25;15(11):e0242711. doi: 10.1371/journal.pone.0242711 (PMC7688172; doi:10.1371/journal.pone.0242711)
Supplement: S1 File — (ZIP) [file pone.0242711.s001.zip › Supporting Information ZIP files/Codebook for the data file.docx]

Codebook for the data file « data_file_Table_1.xlsx » (Table 1)

| **Variables names** | **Variable descriptions** | **Codes** |
| --- | --- | --- |
| ID | Identification number | Numeric |
| Country | Country of site study | 1 = Benin  2 = Mali |
| VPH | Subject record number | Numeric |
| M0quQ01A | Age in year | Numeric |
| M0quQ05 | Marital status | 1=Married  2=Cohabitation  2=Divorced, Separated  3=widow  4=Never Married  5=Unknown |
| M0quQ07 | Number of biological children | Numeric |
| M0quQ13 | Age at first sexual intercourse | Numeric |
| M0quQ14 | Age at first paid sex | Numeric |
| M0quQ15 | Main place of work | 1=Home-Base  2=Private home  3=Bar-Based  4=Hotels  5=Street  6=Night club  7=Others |
| M0quQ15AUT | Other place of work precise | Character |
| M0quQ20 | Monthly income | Numeric |
| M0quQ25 | Latest week total number of sexual partners | Numeric |
| M0quQ26 | Number of paying clients, last 7 days of work | Numeric |
| M0quQ28 | Have a boyfriend | 1 = Yes  2 = No  9 = Unknown |
| M0quQ38 | Frequency of condom use with clients | 1=Never  2=Rarely  3=Occasionally; 4=Often  5=Very often  6=Always  7=No relationships during the period  8=Don't know  9=No response |
| M0quQ45 | Use product into vagina | 1=Yes  2 =No  3=Unknown |
| VPH6 | HPV6 positivity | 1=yes  0 = no |
| VPH11 | HPV11 positivity | 1=yes  0 = no |
| VPH16 | HPV16 positivity | 1=yes  0 = no |
| VPH18 | HPV18 positivity | 1=yes  0 = no |
| VPH26 | HPV26 positivity | 1=yes  0 = no |
| VPH31 | HPV31 positivity | 1=yes  0 = no |
| VPH33 | HPV33 positivity | 1=yes  0 = no |
| VPH34 | HPV34 positivity | 1=yes  0 = no |
| VPH35 | HPV35 positivity | 1=yes  0 = no |
| VPH39 | HPV39 positivity | 1=yes  0 = no |
| VPH40 | HPV40 positivity | 1=yes  0 = no |
| VPH42 | HPV42 positivity | 1=yes  0 = no |
| VPH44 | HPV44 positivity | 1=yes  0 = no |
| VPH45 | HPV45 positivity | 1=yes  0 = no |
| VPH51 | HPV51 positivity | 1=yes  0 = no |
| VPH52 | HPV52 positivity | 1=yes  0 = no |
| VPH53 | HPV53 positivity | 1=yes  0 = no |
| VPH54 | HPV54 positivity | 1=yes  0 = no |
| VPH56 | HPV56 positivity | 1=yes  0 = no |
| VPH58 | HPV58 positivity | 1=yes  0 = no |
| VPH59 | HPV59 positivity | 1=yes  0 = no |
| VPH61 | HPV61 positivity | 1=yes  0 = no |
| VPH62 | HPV62 positivity | 1=yes  0 = no |
| VPH66 | HPV66 positivity | 1=yes  0 = no |
| VPH67 | HPV67 positivity | 1=yes  0 = no |
| VPH68 | HPV68 positivity | 1=yes  0 = no |
| VPH69 | HPV69 positivity | 1=yes  0 = no |
| VPH70 | HPV70 positivity | 1=yes  0 = no |
| VPH71 | HPV71 positivity | 1=yes  0 = no |
| VPH72 | HPV72 positivity | 1=yes  0 = no |
| VPH73 | HPV73 positivity | 1=yes  0 = no |
| VPH81 | HPV81 positivity | 1=yes  0 = no |
| VPH82 | HPV82 positivity | 1=yes  0 = no |
| VPH83 | HPV83 positivity | 1=yes  0 = no |
| VPH84 | HPV84 positivity | 1=yes  0 = no |
| VPH89 | HPV89 positivity | 1=yes  0 = no |
| AGE1 | Age in categories | 1 <25  2 =25-29  3=30-34  4=35-39  5=≥50 |
| NATIONALITE | Country of origin | 1=Benin  2=Nigeria  3=Mali  4 =Ghana  5=Others |
| SCOLARITE | Educational level in categories | 1=Uneducated 2=Primary  3 = Secondary or higher |
| ENFANT_BIOLO | Number of biological children in categories | 1=0  2=1  3=2  4=3  5= ≥ 4 |
| ALCOOL | Alcohol consumption in categories | 1= Ever  2= Never |
| DROGUE | Drug use in categories | 1= Ever  2= Never |
| TABAC | Tobacco in categories | 1= Never  2= Less than 10 cigarettes a week  3= Ten cigarettes and more a week |
| AGE_1_RAPPORT_sup | Age at first sexual intercourse in categories | 1= <18  2= >18 |
| AGE_PAYANT | Age at first paid sex | 1= <18  2= ≥18  3=Unknown |
| PLACE_WORK | Main place of work in categories | 1= Home-based  2=Bar-based  3= others |
| DURE_TRAVAIL | Sex work duration in years | Numeric |
| NEW_DUREE2 | Sex work duration in categories | 1= =< 1  2 = 2 – 3  3 = ≥ 4  4 = Unknown |
| PARTENAIRE_TOT | Latest week total number of sexual partners in categories | 1= <5  2= 5 – 14  3 = ≥ 15 |
| CLIENT_PAYANT | Number of clients at the last seven days in categories | 1= <5  2= 5 – 14  3 = ≥ 15 |
| CONDOM_CONSISTANT | Consistent condom use | 1= yes  2= no |
| DOUCH_AV_AP | Used vaginal douching before and after sex | 1= yes  2= no |
| ANTECEDENT_IST | Self-reported STI in last six months | 1= yes  2= no |
| GONO | *N. gonorrhoeae* positivity | 1= yes  2= no |
| CHLAMYDIA | *C. trachomatis positivity* | 1= yes  2= no |
| TRICO_VAG | *T. vaginalis positivity* | 1= yes  2= no |
| VAGINOSE2 | Bacterial vaginosis | 1= Nugent score ≥7  2= Nugent score <7 |
| CANDIDA | *C. albicans positivity* | 1= yes  2= no |
| HIV | HIV positivity | 1= yes  0= no |
| HIGH_RISK | High risk HPV infection | 1= yes  0= no |
| PROB_HIGH_RISK | Probable High risk HPV infection | 1= yes  0= no |
| LOW_RISK | Low risk HPV infection | 1= yes  0= no |
| ANY_HPV | Any HPV infection | 1= yes  0= no |
| Scores1_HR | Score of high HPV infection | 0 = 0 Type HR-HPV  1 = 1 Type HR-HPV  2 = 2 Types HR-HPV  3= 3 Types HR-HPV  4= 4 Types HR-HPV  5= 5 Types HR-HPV  6= 6 Types HR-HPV |
| MULTIPLE_HR | Multiple infection with HR-HPV types ≥ 2 Types HR-HPV | 1= yes  0= no |
| SCORES2_PROB_HR | Score of probable high HPV infection | 0 = 0 Type pHR-HPV  1= 1 Type pHR-HPV  2= 2 Types pHR-HPV  3= 3 Types pHR-HPV  4= 4 Types pHR-HPV |
| MULTIPLE_PROB_HR | Multiple infection with pHR-HPV types ≥ 2 Types pHR-HPV | 1= yes  0= no |
| SCORES3_LR | Score of low HPV infection | 0 = 0 Type LR-HPV  1= 1 Type LR-HPV  2= 2 Types LR-HPV  3= 3 Types LR-HPV 4= 4 Types LR-HPV  5= 5 Types LR-HPV  6= 6 Types LR-HPV  7= 7 Types LR-HPV |
| MULTIPLE_LR | Multiple infection with LR-HPV types, ≥ 2 Types LR-HPV | 1= yes  0= no |
| SCORES4_ENS | Score of any HPV infection | 0= 0 Type  1= 1 Type  2= 2 Types  3= 3 Types  4= 4 Types  5= 5 Types  6= 6 Types 7= 7 Types  8 = ≥ 8 Types |
| IVA_IVL | VIA/VILI positivity | 1= yes  2= no |
| MULTI_INF | Multiple infection with any HPV types, ≥ 2 Types LR-HPV | 1= yes  0= no |
| VACCIN4 | Any quadrivalent-valent vaccine types | 1= yes  0= no |
| SCORES_VACCIN4 | Score of quadrivalent vaccine type detected | 0=0 type  1=1 type  2=2 types  3=3 types |
| VACCIN9 | Any nonavalent-valent vaccine types | 1=yes  0=no |
| SCORES_VACCIN9 | Score of nonavalent vaccine type detected | 0=0 Type  1=1 Type  2=2 Types  3=3 Types  4=4 Types  5=5 Types |
